# Purine-Metabolising Enzymes and Apoptosis in Cancer

**DOI:** 10.3390/cancers11091354

**Published:** 2019-09-12

**Authors:** Marcella Camici, Mercedes Garcia-Gil, Rossana Pesi, Simone Allegrini, Maria Grazia Tozzi

**Affiliations:** 1 Dipartimento di Biologia, Unità di Biochimica, Via S. Zeno 51, 56127 Pisa, Italy; 2 Dipartimento di Biologia, Unità di Fisiologia Generale, Via S. Zeno 31, 56127 Pisa, Italy

**Keywords:** purine salvage, apoptosis, CD73, cN-II, ADA, PNP, HPRT, IMPDH, SAMHD1, MTH1

## Abstract

The enzymes of both de novo and salvage pathways for purine nucleotide synthesis are regulated to meet the demand of nucleic acid precursors during proliferation. Among them, the salvage pathway enzymes seem to play the key role in replenishing the purine pool in dividing and tumour cells that require a greater amount of nucleotides. An imbalance in the purine pools is fundamental not only for preventing cell proliferation, but also, in many cases, to promote apoptosis. It is known that tumour cells harbour several mutations that might lead to defective apoptosis-inducing pathways, and this is probably at the basis of the initial expansion of the population of neoplastic cells. Therefore, knowledge of the molecular mechanisms that lead to apoptosis of tumoural cells is key to predicting the possible success of a drug treatment and planning more effective and focused therapies. In this review, we describe how the modulation of enzymes involved in purine metabolism in tumour cells may affect the apoptotic programme. The enzymes discussed are: ectosolic and cytosolic 5′-nucleotidases, purine nucleoside phosphorylase, adenosine deaminase, hypoxanthine-guanine phosphoribosyltransferase, and inosine-5′-monophosphate dehydrogenase, as well as recently described enzymes particularly expressed in tumour cells, such as deoxynucleoside triphosphate triphosphohydrolase and 7,8-dihydro-8-oxoguanine triphosphatase.

## 1. Introduction

**Figure 1:**
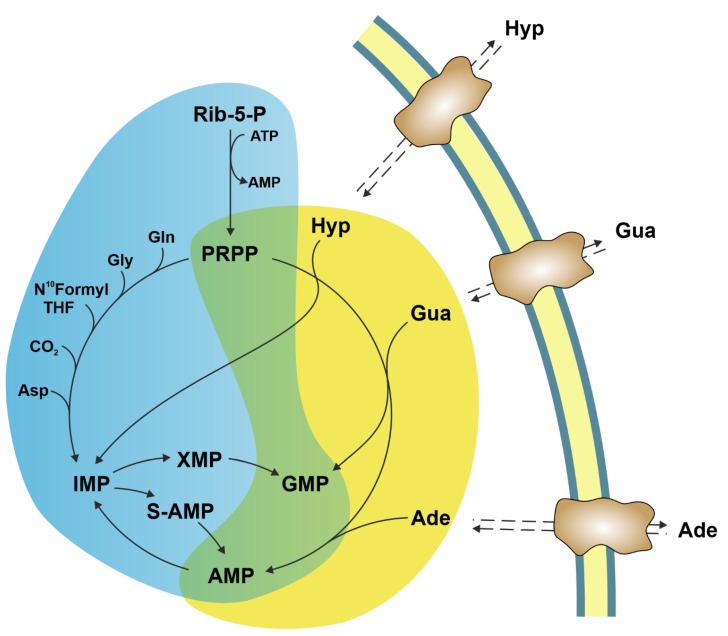
De novo and salvage pathways for purine nucleotide biosynthesis. Cyan background: de novo synthesis; yellow background: salvage synthesis. The figure outlines the central role played by PRPP, needed for both de novo and salvage pathways. Hyp: hypoxanthine; Gua: guanine; Ade: adenine; Rib-5-P: ribose-5-phosphate; PRPP: 5-phosphoribosyl-1-pyrophosphate; Gln: glutamine; Gly: glycine; THF: tetrahydrofolate; Asp: aspartate; S-AMP: succinyl-AMP; XMP: xanthosine-5′-monophosphate; IMP: inosine-5′-monophosphate; GMP: guanosine-5′-monphosphate; AMP: adenosine-5′-monophosphate.

**Figure 2:**
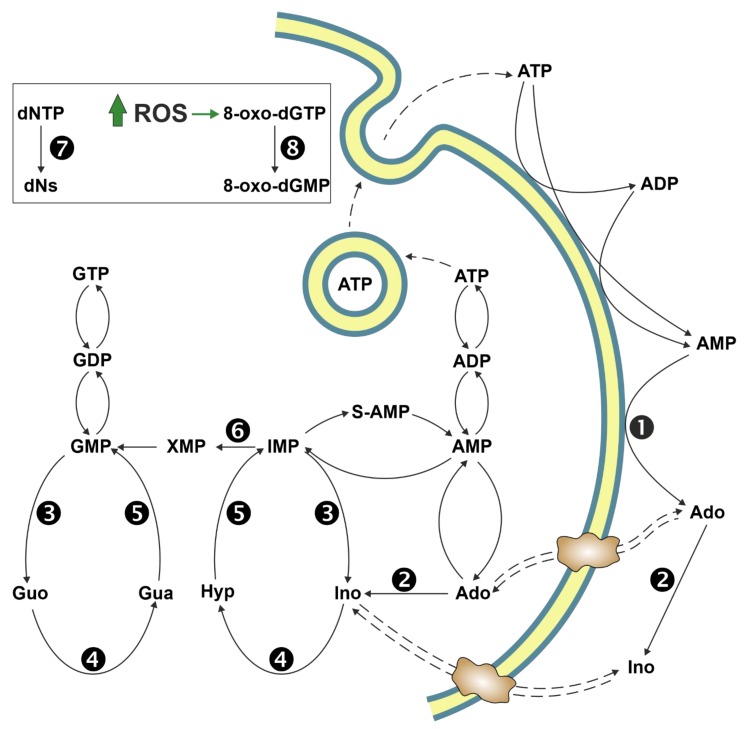
Pathways of purine metabolism. 1: Ectosolic 5′-nucleotidase; 2: Adenosine deaminase; 3: Cytosolic 5′-nucleotidase II; 4: Purine nucleoside phosphorylase; 5: Hypoxanthine-guanine phosphoribosyltransferase; 6: IMP dehydrogenase. Inset: deoxyribonucleoside triphosphates (dNTP) are converted into the respective deoxynucleosides (dNs) by a one-step reaction catalysed by a sterile alpha motif and histidine-aspartate (HD) domain-containing protein 1 (enzyme 7). An increase in reactive oxygen species (ROS) brings about an increase in 8-oxo-dGTP, converted into the monophosphate by human MutT homolog 1 (enzyme 8). Ado: adenosine; Guo: guanosine; Ino: inosine.

Intracellular purine nucleotide concentration is determined and maintained through two distinct pathways, both depending on a common metabolite: 5-phosphoribosyl-1-pyrophosphate (PRPP) (Figure 1). The de novo pathway consists of 10 reactions catalysed by six enzymes, some of which are allosterically regulated mainly by PRPP and purine nucleotides [1]. Furthermore, the six enzymes can cluster near mitochondria and microtubules to form dynamic multienzyme complexes referred to as “purinosomes” [2]. The purinosome formation causes a strong increase in the rate of purine synthesis. This regulatory mechanism ensures that, during proliferation and in the absence of preformed purine ring to be salvaged, the supply of purine compounds is secured. When preformed purine rings are available, they can be converted in one step into the corresponding nucleoside monophosphates through the action of the salvage pathway enzymes adenine phosphoribosyltransferase (APRT) and hypoxanthine-guanine phosphoribosyltransferase (HPRT) which utilize PRPP as a co-substrate (Figure 1) [1]. The availability of preformed purine ring prevents purinosome formation [2]. Regulation both at genetic and protein levels ensures the correct amount of nucleotides to sustain replicative and metabolic needs [3]. Therefore, it is not surprising that alterations of replicative rate and eventually activation of apoptosis are a consequence of dysfunctions of purine metabolism. Furthermore, an imbalance in purine supply can affect also mitochondrial proliferation and function, leading to metabolic changes and apoptosis [4]. Nevertheless, in some cases, the relationship between enzyme dysfunction and proliferation rate, metabolic alterations, and apoptosis is not as simple and direct as expected. The knowledge of the consequences of the alteration of enzymes involved in purine metabolism is essential not only for the understanding of “where and how” these enzymes impact metabolic pathways, but also to uncover whether they can be targets of antineoplastic drugs or be responsible for drug resistance. This review presents the most recent reports on the impact of purine catabolism and salvage enzymes (Figure 2) in the activation of apoptosis and the implied molecular mechanisms. Furthermore, the possible applications of this knowledge to anti-tumour therapy are discussed.

## 2. Ectosolic 5′-Nucleotidase

**Figure 3:**
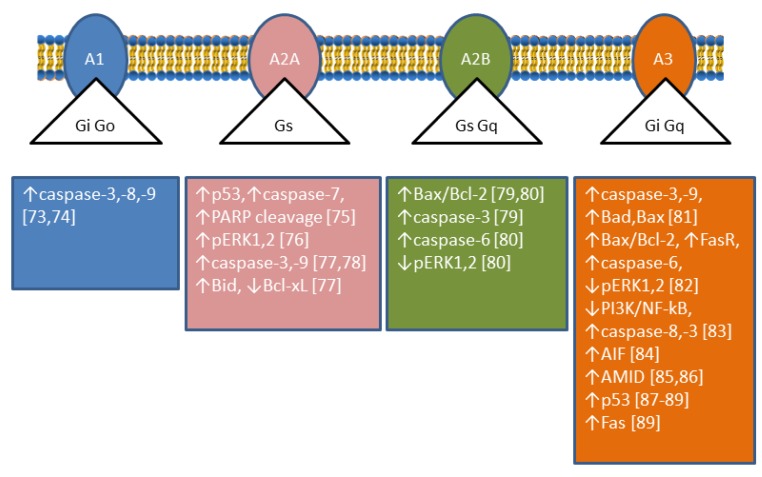
Effectors of adenosine receptor-mediated apoptosis. The figure shows the different types of G-proteins associated with the four adenosine receptors and illustrates the apoptosis effectors found in several models by using agonists and antagonists of the receptors, or receptor silencing. The numbers in brackets refer to the respective reference. Note that adenosine receptors can be involved in survival in other cell types [71].

Ectosolic 5′-nucleotidase (CD73) (EC 3.1.3.5) is a 70-kDa glycosylated protein bound to the outer surface of the plasma membrane by a glycosylphosphatidylinositol anchor. The enzyme is overexpressed in a variety of tumours [5] and in some of them, is associated with a highly invasive cancer phenotype, drug resistance, and tumour-promoting functions [6]. CD73 catalyses the dephosphorylation of extracellular AMP to adenosine (Ado), which plays important roles in many physiological and pathophysiological conditions through G-protein coupled Ado receptors (A1, A2A, A2B, and A3) [7,8]. The roles played by Ado are complex since its interaction with different receptors achieves different results; in particular, A1 and A3 receptors are coupled with Gi-proteins determining a decrease in cyclic AMP (cAMP), while A2A and A2B receptors are coupled with Gs-proteins causing an increase in intracellular cAMP [9] (Figure 3). Therefore, the role played by CD73 depends on the nature and distribution of the Ado receptors in a particular kind of cell. In addition, CD73 plays also many different roles in cell physiology not related to its catalytic activity [10]. Analysing enzymatic and non-enzymatic functions of CD73, it was concluded that both faces were involved in the aggressive behaviour of cancer cells. The enzymatic function seems to be primarily involved in invasion, whereas the non-enzymatic action of the protein contributes to cell adhesion and migration through activation of focal adhesion kinase [10]. Among the tumours in which CD73 is upregulated, breast cancer is the most studied [11]. In some cases of this type of cancer, an involvement of CD73 enzymatic activity was demonstrated, since supply of Ado could be a substitute for enzyme upregulation in promoting proliferation and motility [12]. In other cases, the involvement of different not yet described mechanisms independent of enzyme activity was demonstrated [13]. In all cases, an activation of the Akt/GSK-3β pathway was found to be involved in the tumour growth and motility promoted by CD73 [13]. In human cervical cancer cells, an increase in cell proliferation and motility was associated to CD73 overexpression, but the mechanism was independent of enzyme activity. In fact, CD73 inhibitors were unable to prevent the increase in proliferation in cells that overexpressed the enzyme. In addition, an increase in Ado, that could be expected when the enzyme is overexpressed, induced a decreased cell proliferation [14]. In this type of tumour, an activation of the Akt pathway was demonstrated as well [14]. In colorectal cancer, a downregulation of miR-30a was shown to determine an increase of CD73 expression in tumour cells, which promoted proliferation and inhibited apoptosis. MiR-30a is one of the most important tumour-suppressor factors in various human cancers and its level is significantly decreased in several tumours [15]. Since, as stated before, many effects exerted by the expression of CD73 on tumours are mediated by the participation of the enzyme activity in the conversion of extracellular ATP into Ado, such effects are dependent on the amount and nature of the Ado receptors expressed by tumour cells and other cells present in the tumour microenvironment. In this regard, in contrast to reports for other cancer types (see above), in an in vivo study on medulloblastoma, overexpression of CD73 reduced tumour growth and vascularization, and also promoted differentiation and initiated apoptosis, supposedly by the accumulation of Ado which interacted with A1 receptor [16]. The level of CD73 expression is very important not only for tumour growth and motility, but also for the success of the therapeutic approach. In fact, during chemotherapy, various immunogenic mediators accumulate in the tumour microenvironment, included ATP. Extracellular ATP, in this case, is released by the cells undergoing intrinsically or extrinsically activated apoptosis, through pannexin-1 channels and functions as a “find me” signal for P2Y family receptors expressed by macrophages and dendritic cells [17]. In this mechanism, the rapid ATP degradation catalysed by several extracellular enzymes is a determinant for the immunogenic activation, since the accumulated AMP, which cannot interact with P2Y receptors, can generate Ado through CD73, which mainly mediates the immune-escape of tumour cells interacting with A2A receptors [18]. Moreover, also in the case of regulatory T-cells (Tregs) in a tumour environment, the presence of several signals triggers apoptosis, and apoptotic Treg cells achieved superior immuno-suppression via an oxidative stress-associated mechanism [19]. Therefore, the induction of apoptosis in a tumour environment in which cells highly expressing CD73 and Ado receptors A2A are present is not always a successful therapeutic approach. Recently, CD73 has been targeted for the synthesis of new inhibitory compounds which prevent extracellular Ado formation from AMP and the consequent immune-escape [20].

## 3. Cytosolic 5′-Nucleotidase II

Cytosolic 5′-nucleotidase II (cN-II) (EC 3.1.3.5) is an ubiquitously expressed and highly conserved enzyme that hydrolyses purine nucleoside monophosphates (preferentially IMP and GMP) into their corresponding nucleosides and inorganic phosphate. Its enzymatic activity and biochemical features have been well characterised, and the reader is referred to a recent review for further investigation of these issues [21]. The first observation on its prognostic value in nucleoside analogue-treated patients with acute myeloid leukaemia indicated that patients with high cN-II expression in leukaemic blasts showed a poorer outcome with respect to those with a lower expression [22]. Since then, the implication of cN-II in cancer cells and in the response to anticancer treatment has been extensively demonstrated [23,24]. Indeed, shRNA-based cell models with downregulated cN-II are more sensitive to purine nucleoside and nucleobase analogues, as compared to control cells [25]. The enzyme is highly expressed in tumour cells, and cN-II expression in human neuroblastoma cells and in lung cancer cells correlated with cell proliferation [26,27], whereas its inhibition in human breast cancer cells was associated with a better defence towards reactive oxygen species (ROS) and a better adaptability to glucose deprivation in culture media [28]. In a cell model of lung cancer (A549), an activation of p53 and inactivation of Akt following cN-II partial silencing was demonstrated [27]. Indeed, in an astrocytoma cell line (ADF), transitory cN-II silencing was followed by caspase-3 activation and apoptosis [29]. Furthermore, the siRNA-mediated inhibition of cN-II expression in murine skeletal muscles induced an increase in the AMP/ATP ratio and a subsequent activation of AMP-activated protein kinase (AMPK) [30], even though this was not confirmed in cN-II deficient mice [31]. In parallel to these biological studies, several genetic as well as genome wide association studies have identified the cN-II encoding gene *NT5C2* or some genetic variants as being associated to various pathological conditions such as hereditary spastic paraplegia 45, psychiatric disorders, a disturbance of blood pressure, and a decrease in body mass index [32]. Fluctuation of cN-II expression has been demonstrated to influence the concentration of intracellular nucleotides, depending on the type of cell and the technique utilised for enzyme silencing. In some cases, the alteration of nucleotide concentration is not statistically significant, but the effect on metabolic and proliferative features is still present [27,31,33,34]. These observations indicate that cN-II regulates several cellular pathways through a mechanism at least partially independent of its impact on intracellular nucleotide concentration.

## 4. Adenosine Deaminase

Adenosine deaminase (ADA) (E.C.3.5.4.4) catalyses the deamination of Ado and deoxyadenosine (dAdo) to inosine and deoxyinosine, respectively. There are two isoenzymes of ADA in human tissues, ADA1 and ADA2 [35]. ADA1 is ubiquitous, has a similar affinity for both substrates, and can interact with membrane proteins such as the dipeptidyl dipeptidase-4 (CD26), A1 [36] and A2A receptors [37]. ADA1 also acts as an ectoenzyme which catalyses the deamination of Ado and dAdo in the extracellular space. ADA2 is the main ADA isoenzyme found in human serum [35]. Low ADA activity was found in prostate [38] and gastric tumour tissues [39] and in lymphocytes of patients suffering from different pathologies, such as gynaecological [40], renal [41], head and neck [42], and gastric tumours [43,44], as well as Hodgkin’s lymphoma [45]. Low ADA activity in lymphocytes, as outlined later on in this section, could account for the decreased cellular immune function in cancer patients. Conversely, ADA activity was increased in cancerous tissue from breast [46,47,48], kidney [49], and colorectal tumours [50], in serum of patients with bladder [51] ovarian [52], laryngeal [53], and head and neck squamous cell carcinomas [54,55] and in lymphocytes of patients with chronic lymphocytic leukaemia (CLL) [56]. High ADA activity might be advantageous to the cancer cells by causing, in association with purine nucleoside phosphorylase (PNP), an increase in hypoxanthine, a substrate for the salvage pathway (Figure 2). In addition, increased ADA activity might be a compensatory mechanism against toxic accumulation of its substrates. Indeed, Ado and dAdo are known to induce apoptosis, and ADA inhibition is an antitumoural strategy. Insights from the apoptosis-induced effect of ADA deficiency were obtained from studies regarding severe combined immunodeficiency (SCID) caused by mutations in the *ADA1* gene. The primary cause of lymphotoxicity in ADA-SCID is considered to be the accumulation of dAdo and dATP. In the absence of ADA activity, Ado and dAdo accumulate both in the extracellular compartments and inside the cells. dAdo is then phosphorylated by deoxycytidine kinase (dCK) and/or ADK to dAdo monophosphate, which in turn is converted to dAdo triphosphate (dATP). Intracellular dATP might generate DNA strand breaks and inhibit ribonucleotide reductase, leading to DNA synthesis impairment and apoptosis [57]. Deoxycoformycin (dCF), a powerful inhibitor of ADA [58], has been used alone or in combination with other drugs for the treatment of several types of lymphatic leukaemia [59,60,61,62]. dATP accumulation induced by dCF in hairy cell leukaemia led to activation of p53, release of cytochrome c from mitochondria and activation of apoptotic protease-activating factor 1 (Apaf-1), and therefore caspase-9 and caspase-3 activation [63].

The combination of dAdo and dCF was found to be toxic for several tumoural cell lines such as rat hepatoma cells [64], and human colon carcinoma cell lines LoVo and HT29 [65,66,67]. The treatment with dAdo and dCF in combination resulted in the activation of the apoptotic mitochondrial pathway in LoVo, human astrocytoma, and neuroblastoma cell lines [67,68,69,70] with cytochrome c release and caspase-3 activation. Activation of caspase-8, and of both caspase-9 and -8 has also been found in astrocytoma and in neuroblastoma cells, respectively [69,70]. In astrocytoma cells, but not in neuroblastoma cells, a reduction in the production of lactate preceded the effect of dAdo and dCF on cell viability, suggesting a decreased glycolytic capacity. In both cell lines, dAdo must be phosphorylated in order to exert its cytotoxic effect; however, a decrease in the energy charge was observed in astrocytoma, but not in neuroblastoma cells [69,70].

The involvement of the four Ado receptors in apoptosis of cancer cells has been reported and reviewed recently [71,72]. Apoptosis can occur through A1 [73,74,75], A2A [76,77,78], A2B [79,80], and A3 [81,82,83,84,85,86,87,88,89] receptors (Figure 3). Extracellular Ado can enter the cells through Ado transporters, and as intracellular Ado is converted by ADK to AMP which can activate AMPK, an energy sensor of the cells involved both in survival and cancer suppression (for a recent review see [90]). AMPK is responsible for apoptosis in some human gastric [91,92] cancer cells and astrocytoma cells among others [74]. In human mesothelioma cells this pathway led to upregulation of p53 [93]. p53 exerts its pro-apoptotic effect by transcription-dependent and transcription-independent actions. The targets of p53 transactivation are pro-apoptotic members of the Bcl-2 family (Bax, Bid, Puma, Noxa), as well as other apoptotic effector proteins (Apaf-1, caspase-8, caspase-6), cell death receptors, and cell death ligands. The p53 protein also acts directly in the mitochondria facilitating the oligomerization of Bax and Bak and interacting with anti-apoptotic Bcl-2, Bcl-xL, and Mcl-1 proteins [94]. The ADA inhibitor erythro-9-(2-hydroxy-3-nonyl)adenine (EHNA) but not dCF, induced apoptosis in malignant pleural mesothelioma [95] by increasing intracellular Ado that needed to be converted to AMP, since ADK inhibition neutralised the effect. EHNA also inhibits cyclic nucleotide phosphodiesterase [96], therefore, it was postulated that this enzyme might have a role in the cytotoxicity observed in these cells. In addition, Ado-induced apoptosis involves the intrinsic and extrinsic pathways resulting in caspase-3 activation, and caspase-independent pathways leading to accumulation of Apoptosis Inducing Factor (AIF) (or its homologous AIF-homologous mitochondrion-associated inducer of death, AMID) in the nucleus [71,97]. AIF can be released from mitochondria, migrates to the nucleus where it can recruit nucleases, or organize a DNA-degrading complex [98]. In human hepatoma cells, Ado upregulated AMID, and promoted its translocation to the nucleus where it may induce DNA degradation [99]. Ado accumulation also reverses the action of S-adenosylhomocysteine hydrolase, increasing S-adenosylhomocysteine, which inhibits the transmethylation reactions. S-adenosylhomocysteine has been suggested to be involved in Ado-induced apoptosis in hepatoma HepG2 by altering gene expression [100]. It is interesting to note that dCF, through Ado receptor-dependent mechanisms, was also able to decrease the aggressiveness of cancer cells by modulating migration and invasion and by regulating endothelial cell permeability [101].

## 5. Purine Nucleoside Phosphorylase

Human purine nucleoside phosphorylase (hPNP) (EC 2.4.2.1), an ubiquitously expressed homotrimer, catalyses the phosphorolysis of 6-oxo-ribo- and 6-oxo-2′-deoxyribo-nucleosides to the corresponding bases and pentose-1-phosphate, but does not accept Ado and dAdo as substrates [102]. Conversely, the homoexameric *Escherichia coli* PNP (ePNP) efficiently acts on adenine-based nucleosides [103].

The importance of hPNP for T-cell functions was recognised more than 40 years ago [104] with the discovery that an inherited deficiency of this enzyme caused a severe T-cell lymphopaenia. In fact, the inhibition of PNP allows for the phosphorylation of 6-oxo-2′deoxyribo-nucleosides which are channelled towards nucleotide synthesis instead of degradation. In particular, T-cell specific toxicity results from the inherently high phosphorylation of dGuo and the slow catabolism of dGMP in this cell type [105,106]. The major kinase activities responsible for the phosphorylation of dGuo appear to be cytosolic dCK and mitochondrial deoxyguanosine kinase [106]. dGMP is then converted to dGTP in two non-rate limiting kinase reactions. The overproduction of dGTP perturbs the deoxyribonucleotide pool, inhibits DNA synthesis, and induces cell death through mechanisms that will be discussed later on in this section. These observations provided a rationale for the development of hPNP inhibitors for the treatment of leukaemia. Among these inhibitors, forodesine (also known as immucillin H), a transition state analogue inhibitor of hPNP [107], has been thoroughly investigated as antineoplastic agent, in particular against T-cell mediated disorders. In PNP-deficient mice the mitochondrial deoxyguanosine kinase activity appears to be the responsible for the accumulation of dGTP in mitochondria, which leads to cell death with mitochondrial damage [108]. This abnormal accumulation interferes with mitochondrial DNA synthesis and repair [109], leading to p53 activation and apoptosis [110,111], possibly related to forodesine-induced ROS production and loss of mitochondrial membrane potential [112]. In vitro experiments performed with cultured human leukaemia cells demonstrated the toxicity of the combination of forodesine and dGuo [113,114]. In CLL, results indicate that forodesine induces apoptosis both through a p53-dependent [113] and a p53-independent [112] pathway. Balakrishnan et al. [113] reported studies carried out in leukaemic lymphocytes obtained from patients with CLL. The treatment with dGuo and forodesine in combination led to an increase in dGTP content, which correlated with a stabilization through Ser15 phosphorylation of p53, with an upregulation of downstream protein p21. A correlation between dGTP accumulation and caspase-8, -9, and -3 activation and subsequent polyADP-ribose polymerase (PARP) cleavage was also observed. Furthermore, all these effects appeared to be tumour-specific, as normal T and B lymphocytes isolated from healthy subjects did not undergo apoptosis. Alonso et al. [112] showed that forodesine in combination with dGuo induced a dose-dependent cell death in CLL cells, but the response was independent of deletions in 17p13 (TP53) and 11q22-q23 (ATM), genetic aberrations acquired in advanced disease and associated with drug resistance and short survival [115]. Therefore, forodesine activated the mitochondrial apoptotic pathway, acting by a p53-independent mechanism in tumours with no functional p53. Interestingly, the authors found an upregulation of the p53 homolog p73 at transcriptional and translational level in CLL cells with p53 deletion, thus suggesting that this antileukaemic agent might be beneficial in CLL patients with impaired p53-dependent apoptotic pathway [115]. For the clinical practice of forodesine against lymphoblastic leukaemia, the reader is referred to previously published reviews and articles [116,117,118].

**Figure 4:**
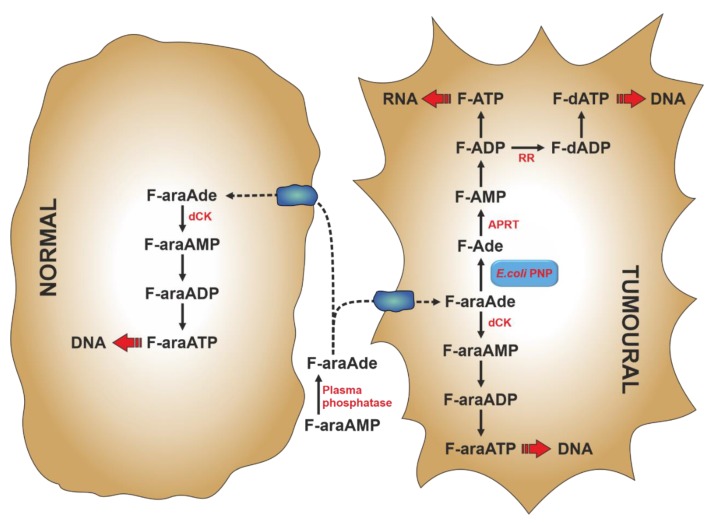
Metabolism of F-araAMP in normal and tumoural cells subjected to *Escherichia coli* PNP (ePNP)/F-araAMP GDEPT. 9-β-arabinofuranosyl-2-fluoroadenosine 5’-monophoaphate (F-araAMP) is cleaved by plasma phosphatases into 9-β-arabinofuranosyl-2-fluoroadenine (F-araAde), which enters both normal and tumoural cells. Inside the cell, it is activated to the monophosphate by cellular deoxycytidine kinase (dCK). In tumour cells, the presence of ePNP allows for an additional activation pathway, which proceeds through a phosphorolytic cleavage to give 2-fluoroadenine (F-Ade), which is activated by cellular adenine phosphoribosyltransferase (APRT). Through the action of ribonucleotide reductase (RR), F-ADP is converted to the respective deoxynucleotide, thus interfering also on DNA synthesis. GDEPT: gene-directed enzyme prodrug therapy.

Gene-directed enzyme prodrug therapy (GDEPT) is a strategy of prodrug delivery which comprises a three component-system: an inactive drug (prodrug), a gene coding for the enzyme that converts the inactive prodrug into the active drug, and a vector [119]. For the choice of the vector and the specific gene delivery to the target tumour cell, the reader is referred to previously published reports [119,120]. In the early 1990s Parker et al. developed a gene therapy strategy based on the selective expression of the ePNP gene in tumour cells [121,122]. The bacterial enzyme, unlike mammalian PNP, can accept Ado and its analogues as substrates [103] and can be used to cleave nontoxic purine nucleoside analogues to very cytotoxic adenine analogues [122]. The toxic purine base analogues generated by ePNP readily diffuse across cell membrane, therefore killing not only the tumour cell in which they are generated, but also many surrounding tumour cells that do not express ePNP (bystander effect). The first prodrug used with ePNP was 9-β-d-[2-deoxyribofuranosyl]-6-methylpurine (MeP-dR), which, being a poor substrate for human salvage enzymes, such as dCK and hPNP, is not cytotoxic to human cells [123]. Therefore, its toxicity is exerted in tumour cells, where ePNP GDEPT is directed: in fact, 6-methylpurine (MeP), the product of MeP-dR phosphorolysis, is activated to the cytotoxic compound by cellular HPRT and/or APRT activities (see Section 6) and then incorporated both in RNA and DNA. Two other dAdo analogues, 2-fluoro-2′-deoxyadenosine (F-dAdo) and 9-β-arabinofuranosyl-2-fluoroadenine (F-araAde), have also received attention as substrates of ePNP. They are transformed by ePNP to 2-fluoroadenine (F-Ade), an adenine analogue approximately 100-fold more potent than MeP [122], which is converted to the cytotoxic mononucleotide by APRT. With F-araAde being an insoluble compound, it is administered as F-araAde-5′-monophosphate (F-araAMP), which must be dephosphorylated by plasma phosphatases, before entering the cell (Figure 4). An important feature of the ePNP GDEPT is its inhibition of protein and RNA/DNA synthesis, making it a good therapeutic approach for solid tumours, which exhibit a high fraction of non-replicating cells [123,124,125]. The ePNP/F-araAMP GDEPT in combination with docetaxel was used against multidrug-resistant ovarian cancer cells [126] and castration-resistant prostate cancer cells [127]. In both cases the involvement of apoptosis in tumour cell death was established. In particular, in ovarian cancer cells, a down-regulation of anti-apoptotic Bcl-2 protein and upregulation of pro-apoptotic Bax, Bik, and Bok proteins were achieved with the treatment. Furthermore, a caspase-mediated proteolysis of PARP was observed [126]. Krohne et al. [128] demonstrated that ePNP/F-araAMP induced hepatocellular carcinoma cell death predominantly by apoptosis, which occurred earlier in p53-positive HepG2 cells, as compared to p53-negative Hep3B cells. However, the efficiency of tumour cell death was similar in both cell lines. Moreover, they also demonstrated that the induction of apoptosis was independent of the Fas/FasL signalling pathway. They concluded that the ePNP GDEPT could be advantageous against tumours carrying p53 mutations or resistant to Fas-mediated apoptosis [128].

## 6. Hypoxanthine Guanine Phosphoribosyltransferase

HPRT (EC 2.4.2.8) plays a central role in the purine salvage pathway. The enzyme catalyses the conversion of guanine and hypoxanthine to the respective nucleoside monophosphates, by using PRPP as donor of the phosphoribosyl moiety [129]. Adenine is not a substrate of the enzyme; indeed, for the salvage of this purine base, an additional APRT activity (EC 2.4.2.7) is required [130]. The relevance of HPRT in the process of activation of thiopurines for the treatment of malignancies, rheumatic diseases, inflammatory bowel disease, and other pathologies is well documented [131,132]. In fact, HPRT catalyses the conversion of 6-thioguanine to 6-thioguanosine monophosphate and represents the first step towards the production of deoxy-6-thioguanosine 5′-triphosphate. This modified deoxynucleoside triphosphate is incorporated into DNA and is further methylated by S-adenosylmethionine to form S6-methylthioguanine, which, during DNA replication, pairs with thymine and its normal partner, cytosine [133]. The mispairs induced by 6-thioguanine are processed by the DNA mismatch repair leading to cell-cycle arrest followed by apoptosis [133,134]. In colorectal cancer cell lines proficient in mismatch repair, an interconnection between autophagy and apoptosis has been demonstrated. In fact, thiopurines induced autophagy as a survival mechanism which antagonises apoptosis. On the other hand, apoptosis was the major cell death mechanism, since caspase inhibition protected the cells from death induced by thiopurines. Interestingly, the inhibition of apoptosis promoted autophagic response [135]. Thus, a tight interplay between apoptosis and autophagy controls cell fate in response to thiopurines. In fact, thiopurines induced mitochondrial depolarization and increased ROS production, which activated mitophagy for the degradation of damaged mitochondria. However, when mitochondrial depolarization exceeded the threshold for the activation of the cell death machinery, cells were directed to apoptotic death [135]. Therefore, ROS play a relevant role in thiopurine-induced cell death and represent a critical factor that links autophagy and apoptosis through mitochondria.

## 7. Inosine 5′-Monophosphate Dehydrogenase

**Figure 5:**
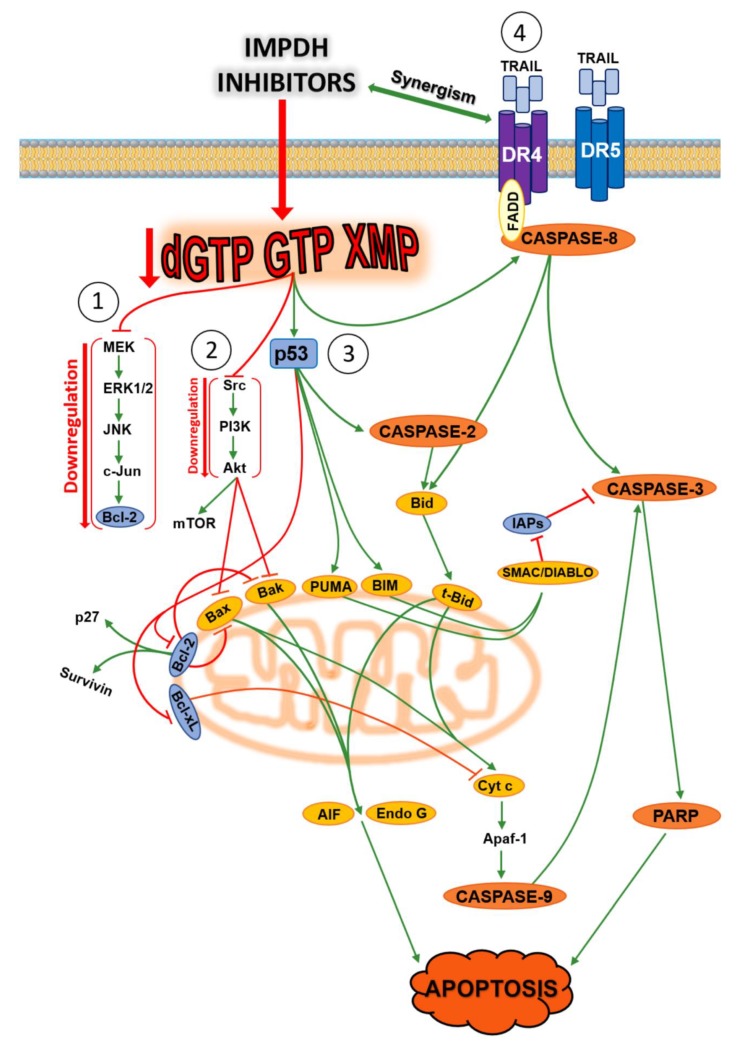
Apoptotic pathways triggered by inosine 5′-monophosphate dehydrogenase (IMPDH) inhibitors. The decrease in guanylate pool can trigger apoptosis through multiple pathways: (**1**): downregulation of the MEK/ERK pathway with inhibition of Bcl-2 and activation of Bax and cytochrome c release [167]; (**2**): downregulation of Src/PI3K pathway with inhibition of Akt, with downregulation of mammalian target of rapamycin (mTOR) and activation of pro-apoptotic Bak and Bax with Apoptosis Inducing Factor (AIF) and endonuclease G (Endo G) release from mitochondria (caspase-independent apoptosis) [136,162,163]; (**3**): upregulation of p53 with (a) downregulation of Bcl-2 and Bcl-xL, with consequent inhibition of p27 and survivin, cytochrome c (Cyt c) release, and activation of caspase-9, caspase-3 and polyADP-ribose polymerase (PARP; intrinsic apoptotic pathway) [159,160,161,164,165], (b) upregulation of PUMA and BIM with consequent SMAC/DIABLO release from mitochondria, inhibition of Inhibitor of Apoptosis (IAPs) (a caspase-3 inhibitor) with activation of caspase-3 [164], and (c) activation of caspase-2 with cleavage of Bid into truncated Bid (t-Bid) and AIF/Endo G release from mitochondria [164]; (**4**): synergistic effect of IMPDH inhibitors with TRAIL through binding with death receptors (DR4 and DR5) which recruit initiator caspase-8 via the adaptor protein FADD. Activated caspase-8 stimulates apoptosis via two parallel cascades: direct cleavage and activation of caspase-3, or cleavage of Bid into t-Bid which translocates to mitochondria, inducing cytochrome c release, with sequential activation of caspase-9 and -3 (extrinsic apoptotic pathway) [164]. TRAIL: tumour necrosis factor-related apoptosis-inducing ligand; FADD: Fas-associated protein with death domain.

Inosine 5′-monophosphate dehydrogenase (IMPDH) (EC 1.1.1.205) is a cytoplasmic enzyme that catalyses the NAD-dependent oxidation of inosine monophosphate (IMP) to xanthosine monophosphate (XMP), the first and rate-limiting step toward the de novo synthesis of GTP [136]. This enzyme plays a central role in purine metabolism, since guanylates are needed not only for nucleic acid synthesis, but also for cellular regulation, such as signal transduction, energy transfer, and microtubule dynamic instability [137]. Two isoforms of IMPDH have been identified: type 1 and type 2 [138]. Human IMPDH type 1 (hIMPDH1) is ubiquitously expressed and its activity is maintained at near constant level in normal and neoplastic cells, whereas the human IMPDH type 2 (hIMPDH2) isoform appears upregulated in proliferating cells [139]. The disproportionate increase of hIMPDH2 activity in neoplastic cells has made this isoform a key target for the development of anticancer drugs. The underlying rationale for the increased activity of hIMPDH2 may be that, although guanylates can be salvaged from guanine by HPRT, the level of circulating guanine is low in dividing cells and this route is probably insufficient to satisfy the needs of guanylates. Indeed, both natural and synthetic inhibitors of IMPDH are used as anticancer [139], antiviral [140], immunosuppressive, and antimicrobial agents [141,142,143,144,145,146]. In Tuberous Sclerosis Complex 2 (TSC-2) deficient cell and tumour models with aberrantly elevated mammalian Target of Rapamycin Complex 1 (mTORC1) signalling and actively producing ribosomes, IMPDH inhibition, by limiting the nucleotide pool, leads to selective replication stress, DNA damage, and apoptosis [147]. Moreover, a selective vulnerability to IMPDH inhibition was reported in a subset of small cell lung cancers, expressing high level of MYC, a transcription factor which appears to regulate purine biosynthesis by activating transcription of *IMPDH* [148]. Recently, it was found that un upregulation of IMPDH2 drives GTP biosynthesis, enhances rRNA and tRNA synthesis, and stimulates nucleolar hypertrophy in glioblastoma cells, thus connecting these features of malignant transformation [149]. Several research groups reported the non-enzymatic functions of IMPDH; IMPDH binds to DNA and RNA in vivo, in a manner independent of its catalytic activity [150]. Indeed, IMPDH was found in the nuclei of human cells, and several experiments suggest that IMPDH has a role in replication, transcription and translation. For example, IMPDH interacts with polyribosomes through a subdomain and is associated with translating rhodopsin mRNA [151]. Moreover, the evidence that recruitment of IMPDH to actively transcribed genes mediates C-terminal domain phosphorylation of RNA polymerase II in yeast supported a new cellular function of IMPDH [152]. IMPDH also acts as a cell- cycle-regulated transcription repressor. At the end of S-phase, the enzyme accumulates in the nucleus and represses proliferation genes such as those coding histones and E2f, a master driver of the G1/S transition [153]. In human embryonic kidney HEK293 and human colon cancer HCT116 cells, p53 regulated purine metabolism through a p53-miR-34a-IMPDH pathway (miR-34a is a master regulator of tumour suppression) and consequently, miR-34a-mediated inhibition of IMPDH perturbed the GTP-dependent Ras signalling pathway [137]. Inhibition of IMPDH activity has also been shown to induce differentiation in some cancer cell lines [154,155,156,157], while apoptosis was induced in other cell lines [158,159,160,161]. Over the past 30 years, several IMPDH inhibitors have been tested on many cancer models. Apoptosis triggered by these inhibitors occurs through multiple pathways depending on the type of compound and the used tumoural model. Figure 5 summarizes the apoptotic pathways triggered by the best known IMPDH inhibitors [136,137,162,163,164,165,166,167,168,169,170]. In all the experiments, apoptotic mechanisms were reversed by the simultaneous addition of guanosine, that, through the salvage pathway, circumvents the IMPDH inhibition by restoration of the guanylate pool.

## 8. Sterile Alpha Motif and HD Domain-Containing Protein 1

Sterile alpha motif and HD domain-containing protein 1 (SAMHD1) was identified as a mammalian deoxynucleoside triphosphate triphosphohydrolase (dNTPase, EC 3.1.5.-), which plays a key role in the regulation of dNTP homeostasis through hydrolysis of intracellular dNTPs to give a free deoxynucleoside and a triphosphate [171,172]. SAMHD1 has been also identified as an important host restriction factor that inhibits the infection of several retroviruses and DNA viruses by diminishing the intracellular dNTP pool needed for their replication [173]. SAMHD1 has also been implicated in the regulation of immune responses [174]. Homozygous mutations in *SAMHD1* gene have been identified in 17% of patients with Aicardi-Goutières syndrome (AGS), an autoimmune disorder that is attributed to excessive accumulation of small DNA fragments in brain. Additionally, homozygous deletion in the *SAMHD1* gene was also identified in atypical AGS patients [175]. *SAMHD1* somatic mutations have been identified in several cancers, including solid cancers such as glioblastoma [176], colorectal [177], lung [178], and pancreatic cancers [179] and blood-related malignancies such as CLL [180] and myeloma [181]. Additionally, SAMHD1 mRNA and/or protein expression is also significantly downregulated in CLL [180] and breast [182] and lung cancers [183]. Crystallographic studies have demonstrated that SAMHD1 is a homotetrameric enzyme allosterically activated by dNTPs [184]. Each monomer contains one catalytic site specific for dNTPs and two allosteric sites: site one has a very high affinity for GTP and dGTP and site two is specific for deoxyribose and binds all four dNTPs with lower affinity than site one [185]. The active site of SAMHD1 can accommodate various base modifications, indeed, SAMHD1 hydrolyses 2-amino-2′-dATP, O6-methyl-dGTP, 5-methyl-2′-dCTP and 2-thio-dTTP as well as the canonical substrates, suggesting a role of the enzyme in the metabolism of nucleoside analogues of therapeutic interest [186]. Furthermore, monomeric and dimeric SAMHD1 bind to single-stranded nucleic acids [187,188], while the tetrameric form of the enzyme is active as a dNTPase [188]. On this basis, Seamon et al. [188] proposed a model in which the level of cellular dNTPs operates as a switch between the two types of SAMHD1 activity. Under conditions of low dNTP concentrations, the monomeric and dimeric forms would prevail, with high affinity for single-stranded RNA, whereas when dNTP concentrations are high, the tetrameric dNTPase would be predominant. These observations suggest that the single-stranded nucleic acid binding activity of SAMHD1, in addition to its dNTPase action, are likely determinants involved in retrovirus restriction and immune activation. Recent progress indicates that gene mutations and epigenetic mechanisms lead to downregulation of SAMHD1 activity or expression in multiple cancers. Impaired SAMHD1 function can cause increased dNTP pool resulting in genomic instability and cell-cycle progression, thereby facilitating cancer cell proliferation [189]. In line with this conclusion, exogenous expression of SAMHD1 inhibited proliferation and induced apoptosis in cutaneous T-cell lymphoma-derived HuT78 cells, through increased activation of extrinsic apoptotic signalling mediators, and sensitised the cells to FasL-stimulated apoptosis [190]. In addition, in an acute myeloid leukaemia-derived human leukaemic cell line (THP I), *SAMHD1* knockout induced cell proliferation and reduced apoptosis and this effect was in part due to activation of PI3K-Akt-p27 signalling axis. Furthermore, the *SAMHD1* knockout attenuated the ability of THP-1 cells to form subcutaneous tumour cells in xenografted immunodeficient mice. This effect correlated with significantly increased expression of tumour necrosis factor α (TNF-α) in cancer, which may suggest that TNF-α-mediated inflammation could account for the decreased tumourigenicity in vivo [191].

## 9. Human MutT Homolog 1

**Figure 6:**
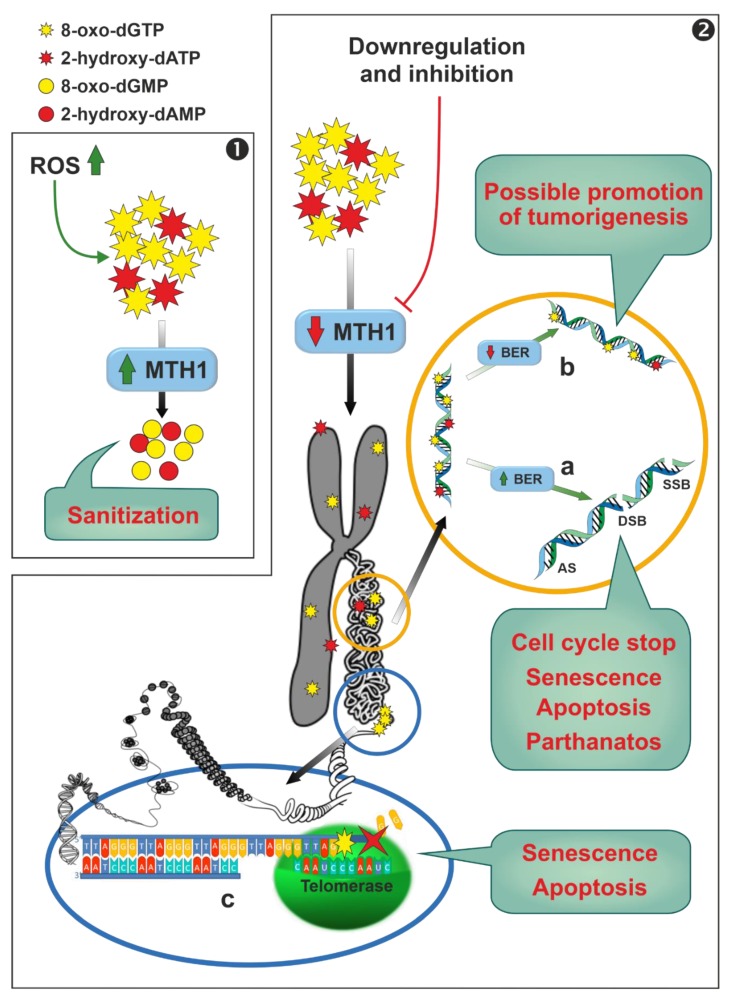
Scheme of possible effects of therapeutic use of human MutT homolog 1 (MTH1). Panel 1: Cancer cell proliferation is stimulated by the accumulation of ROS, which favours the formation of 8-oxo-dGTP and 2-hydroxy-dATP. In order to avoid the potentially toxic effects of the incorporation of oxo-derivatives of nucleotides, the cells upregulate MTH1 sanitizing enzyme. Panel 2: downregulation or inhibition of MTH1 leads to an abnormal incorporation of oxidised nucleotides in DNA. Panel 2a: in order to deal with this excessive accumulation of oxo-nucleotides in DNA, the base excision repair (BER) system is, in general, upregulated. If the misincorporation of oxo-nucleotides exceeds the capability of BER, abasic sites (AS) or single strand brakes (SSB) or double strand brakes (DSB) accumulate, leading to several serious consequences. Panel 2b: if BER is downregulated, incorporated oxo-nucleotides are not repaired. In this case no cytotoxic effect is reported, but a promotion of tumourigenesis can occur. Panel 2c: the misincorporation of 8-oxo-dGTP by telomerase into the repeated telomeric sequence TTAGGG leads to the inhibition of the polymerase activity of the enzyme.

Human MutT homolog 1 (MTH1) is a widely expressed monomeric enzyme that hydrolyses oxidised purine nucleoside triphosphates, such as 8-oxo-dGTP, 8-oxo-dATP and 2-hydroxy-dATP, to monophosphates and pyrophosphate (7,8-dihydro-8-oxoguanine triphosphatase, EC 3.6.1.55) [192]. It is mostly present in cytosol and, in lesser amounts, in mitochondria and in nuclei. Higher expression levels of the enzyme were found in thymus, testis, embryo, proliferating blood lymphocytes [193], and in cancer cells when compared with normal ones [194]. Its enzymatic activity, together with its cellular distribution and the overexpression in cancers, lead to hypothesise for MTH1 a possible role in the sanitization of nucleotide pools both for nuclear and mitochondrial DNA/RNA replication and transcription processes, preventing the misincorporation of oxidised nucleotides into nucleic acids (Figure 6, Panel 1). In fact, cancer cells are characterised by a marked alteration of the redox status with a consequent increase in ROS [195]. A small increase in ROS levels promotes proliferation and cancer cell survival [196,197], whereas large amounts of ROS inhibit proliferation [198] and promote senescence [199,200] and apoptosis in several cancer models [200,201,202,203]. While it is widely accepted that MTH1 is dispensable in healthy cells [194], the role of the enzyme in removing oxidised dNTP in cancer cells, thus promoting their survival, and the consequent possibility to use this enzyme as a chemotherapeutic target, is controversial. These discordances, arising probably from the use in the different reports of different experimental conditions (inhibitors, siRNA, shRNA, or CRISPR technique) and models (cell cultures, xenograft, etc.), have been reviewed in the past few years and the reader is referred to these extensive reviews for a close examination [194,204,205,206]. We will focus on the mechanisms by which targeting MTH1 could lead, according to the supporters of this thesis, to apoptosis and/or senescence of cancer cells. Downregulation or inhibition of MTH1 leads to the incorporation in nuclear and mitochondrial DNA of oxidised nucleotides (Figure 6, Panel 2), which may be removed by a base excision repair (BER) process [194,207]. It has been proposed that wild type p53 is able to stimulate the BER response at different levels [208,209,210]. However, the excessive incorporation in DNA of oxidised nucleotides, may cause an overload of the finely coordinated BER system, leading eventually to the production of either abasic sites or single strand brakes or double strand brakes [194]. A connection between this DNA damage and different detrimental effects (stop of cell cycle in G1, senescence, apoptosis p53, caspase-3 mediated or parthanatos) has been proposed [111,194,200,206,211] (Figure 6, panel 2a). Conversely, if the BER process does not work properly, or in models containing null or mutant p53 [212,213], tumourigenesis can be promoted via the accumulation of mutations caused by the mispairing of 8-oxo-dGTP and 2-hydroxy-dATP (Figure 6, Panel 2b). In addition, 8-oxo-dGTP promotes chain termination following insertion by telomerase into the repeated telomeric sequence TTAGGG [214], and in oxidant conditions, cancer cells with very short telomers are quite sensitive to MTH1 downregulation [214] (Figure 6, Panel 2c).

## 10. Concluding Remarks

A correct balance of NTPs and dNTPs is necessary for the prevention of multiple pathologies. A healthy cell must maintain two asymmetric and spatial-temporal dNTP pools; one for nuclear DNA (nDNA) synthesis and repair and another for mitochondrial DNA (mDNA) replication and repair. Disruptions in dNTP balance are associated with enhanced mutagenesis, leading to genomic instability which promotes cancer [215] and may have a role in metabolic disease [216]. Cytosolic dNTP pool concentrations positively correlate with the cell cycle. In fact, the quantity of dNTPs at the beginning of S-phase is not sufficient for a complete DNA duplication [217]. The increase of dNTPs during the S-phase is necessary for faithful nDNA replication. mDNA is replicated continuously in post-mitotic cells, and faithful maintenance of mDNA also depends on correctly balanced dNTPs [218]. Thus, both proliferating and non-proliferating cells need to fine-tune nucleotide and dNTP synthesis to allow for both nDNA and mDNA replication and repair to maintain the health of the cell. Indeed, starvation or mTORC1 inhibition leads to selective autophagy of ribosomes, providing a source of nucleosides to be used by the salvage pathway enzymes for the synthesis of nucleotides, thus contributing to starved cell survival [219]. An imbalance of NTPs may have metabolic consequences and also generate an imbalance of dNTPs that can be synthesised by salvage of deoxynucleosides and by NDP reduction catalysed by ribonucleotide reductase. A plethora of signals concur to activate DNA repair mechanisms or to promote apoptosis in cells in which NTP pools are altered. The knowledge of the pathways linking enzyme expression or inhibition and apoptosis is fundamental to find new chemotherapeutic approaches able to activate apoptosis in cancer cells. Enzyme inhibition or silencing techniques were utilised to demonstrate the involvement of several interconverting or salvage enzymes in the determination of nucleotide imbalance. Some enzymes described in this review have been demonstrated or suspected to also have a role distinct from catalytic activity. In fact, ectosolic 5′-nucleotidase promotes cancer not only through the production of ectosolic Ado: cytosolic 5′-nucleotidase regulates proliferation and apoptosis in a way not so easily attributable to its regulation of intracellular nucleotide concentration, IMPDH also acts as transcription factor, and, finally, SAMHD1 binds to single-stranded RNA. All these observations make it difficult to directly link enzyme dysfunction with NTP imbalance and activation of apoptosis. Unfortunately, in many papers the nucleotide imbalance was inferred by the alteration of enzyme expression or inhibition and the consequent activation of the apoptotic programme, while the effective nucleotide concentration was not measured. Furthermore, in the case of enzymes involved in the Ado metabolism it is not so easy to distinguish between the effects exerted by Ado itself and those mediated by the interaction with its receptors. In addition, dAdo that does not interact with Ado receptors is able to trigger apoptosis through the formation of dATP and possibly through other still unknown mechanisms. In any case, the knowledge of the catalytic capacity of the enzymes involved in the pathways of Figure 2 and of their substrate specificity and regulation opened the way to novel pharmacological approaches to cancer therapy. Furthermore, the sometimes-surprising results obtained by their inhibition or silencing indicate that much more needs to be unravelled about purine salvage and interconverting pathways.
